# Examining Electronic Health Records to Investigate Sex Differences in Oral Diseases and Conditions

**DOI:** 10.1002/cre2.70455

**Published:** 2026-09-08

**Authors:** Emma Fetchko, Linda Sangalli, Ariadne Letra

**Affiliations:** ^1^ University of Pittsburgh School of Dental Medicine Pittsburgh Pennsylvania USA; ^2^ College of Dental Medicine Midwestern University Downers Grove Illinois United States; ^3^ Departments of Oral and Craniofacial Sciences and Endodontics University of Pittsburgh School of Dental Medicine Pittsburgh Pennsylvania USA; ^4^ Center for Craniofacial and Dental Genetics University of Pittsburgh School of Dental Medicine Pittsburgh Pennsylvania USA

**Keywords:** health records, oral diseases, oral health, sex differences

## Abstract

**Objectives:**

Sexual dimorphism has been shown to influence disease predisposition and/or progression, although population‐based studies in dental, oral, and craniofacial (DOC) diseases and conditions are limited. This study aimed to identify sex‐based differences in DOC diseases/conditions using two large health data repositories.

**Methods:**

Retrospective cross‐sectional study of health record data obtained from adult participants (> 18 years old) in the NIH *All of Us* Research Program (*n* = 254,700) and the BigMouth Data Repository (*n* ≈ 4.7 million). The number of males and females presenting each selected condition in each database was recorded. Sex‐specific association analysis for each condition was performed using chi‐square tests (α ≤ 0.0002). Female‐to‐male odds ratio (OR) and confidence intervals were also calculated.

**Results:**

Significant sex‐related differences were found for 61/87 concepts (70%), with 33 concepts (54%) showing female bias and 28 (46%) showing male bias (*p *≤ 0.0002). Analysis of BigMouth data showed sex bias for 90/230 (39%) concepts investigated, of which 87 (97%) showed female bias and 3 (3%) showed male bias (*p *≤ 0.0002).

**Conclusions:**

This study provides evidence of sex bias in numerous oral and dental conditions in the populations studied. Additional studies in other populations might provide further insight into the role of sexual dimorphism in these conditions.

## 1 Introduction

Sex is a biological variable that accounts for differences between males and females across multiple species and cellular domains such as growth, metabolism, reproduction, and aging processes (Manandhar et al. 2018). These differences may confer increased vulnerability for specific infections, diseases, and conditions in one sex over the other (Ubeda and Janse 2017). Previous studies have found significant differences in immune functions between males and females that influence the course and manifestation of infections (Klein 2000). Males typically exhibit a more robust acute inflammatory response, while females display attenuated inflammatory reactions (Valerio and Kirkwood 2018). This divergence is a consistent trait among mammals, where the innate immune response significantly differs between sexes (Valerio and Kirkwood 2018). Moreover, studies have indicated that sex hormones exert differential effects on the immune system, contributing to distinct risk profiles for certain disorders in males and females (Keselman and Heller 2015). In addition, specific genes on the X chromosome are shown to influence metabolism, injury response, behavior (Arnold et al. 2016; Keogh and Boerner 2020; Lipsky et al. 2021), as well as expression of cytokines, and other immune response mediators (Klein and Flanagan 2016). More recently, a growing body of research has highlighted significant differences in disease manifestations between males and females, driven by these underlying biological mechanisms (Mauvais‐Jarvis et al. 2020).

Yet, large clinical studies or studies utilizing human data examining sex‐related implications in oral, dental, and craniofacial diseases and disorders are scarce. Although oral diseases affect almost half of the population worldwide (World Health Organization 2022), the understanding of sex‐specific manifestations that could confer differential risk for certain oral conditions remains limited. These variations in oral manifestations could be attributed to differences in immune function, hormonal influences, or genetic predisposition (Sangalli et al. 2023). Without a clear understanding of which conditions show a different manifestation in males and females, grasping the underlying cause becomes challenging.

Numerous studies have shown contrasting biological traits between men and women, including certain investigations into specific oral conditions and sex‐related distinctions. Some examples include apical periodontitis and dental caries. Studies of apical periodontitis have yielded mixed outcomes, with findings supporting no significant difference between the sexes, while others suggesting a higher prevalence in women (Al‐Nazhan et al. 2017; Berlinck et al. 2015). Similarly, studies focused on dental caries have conflicting findings, with some indicating female predilection (Lukacs and Largaespada 2006; Lukacs 2008, 2011), others favoring males (Furuta et al. 2010), and others failing to demonstrate any sex‐related differences (Shaffer et al. 2015). Discrepancies among these results are likely due to differences in sample sizes, parameters evaluated, and populations studied. Therefore, additional studies with large sample sizes are warranted to determine the oral and dental conditions likely affected by sexual dimorphism.

The National Institute of Health (NIH) *All of Us* Research Program (*All of Us*) (The All of Us Research Program Investigators 2019) consists of a national effort to collect medical, dental, and sociodemographic data, as well as biological samples from one million people in the United States to foster advancements in precision medicine and improve health. Through the *All of Us* Researcher Workbench, investigators can access electronic health record (EHR), survey, and genomic data of program participants in a completely de‐identified format to conduct research. It constitutes a valuable publicly available tool for conducting studies, including a large sample size, aimed at enhancing personalized medicine approaches (Ramirez et al. 2022). Researchers can query the database for specific disease/condition concepts and obtain data on the total number of participants presenting with each concept, their male‐female distributions, race/ethnicity, and age ranges. Researchers may be approved to use *All of Us* data after signing a data use agreement and having completed research ethics training modules. Similarly, the BigMouth Dental Repository (BigMouth) is a centralized dental data repository derived from a national consortia of US dental schools (Consortium of Oral Health Research and Informatics, COHRI) (Walji et al. 2014). Launched in 2012, BigMouth contains de‐identified EHR data obtained from dental diagnostic and procedure codes, as well as self‐reported medical history data from patients seen at each of the current 11 participating dental schools. BigMouth is not publicly available; access is provided to those institutions that contribute to the repository. While *All of Us* and BigMouth have inherent differences due to their sample population and types of data available, both databases offer immense potential for research in dental, oral, and craniofacial (DOC) research and evidence‐based dentistry on a large scale.

In this study, we leveraged the availability of large‐scale data from *All of Us* and BigMouth repositories to investigate common DOC diseases and conditions associated with sexual dimorphism. The results highlight numerous conditions for which sexual dimorphism may play a role in the pathogenesis and provide insight into the development of future precision‐based diagnostic and treatment strategies.

## 2 Materials and Methods

### 2.1 Data Sources

This retrospective cross‐sectional study used medical/dental record data from adult participants (aged 18‐89 years old) obtained via the NIH *All of Us* Research Program (*n* = 254,700) and the BigMouth Dental Data Repository (n ≈ 4.7 million). Data search was conducted by a single investigator (EF) between May and August 2023. The study adhered to the criteria outlined in the STrengthening the Reporting of OBservational studies in Epidemiology (STROBE) guidelines (von Elm et al. 2008).

A comprehensive set of search terms was built around common DOC diseases and conditions, which we further refer to as DOC concepts. For *All of Us*, search terms were informed by the Systemized Nomenclature of Medicine (SNOMED, https://www.snomed.org/) identification number or International Classification of Diseases (ICD)‐10 (https://www.cdc.gov/nchs/icd/icd10.htm), and included “oral carcinoma,” “oral cancer,” “tongue cancer,” “oral tumor,” “periodontitis,” “periodontal disease,” “dental,” “dental disease,” “tooth,” “oral,” “mouth,” “infection,” “caries,” “dental anomaly.” The same search terms were utilized for the BigMouth queries to identify diagnostic codes associated with each concept.

**Figure 1:**
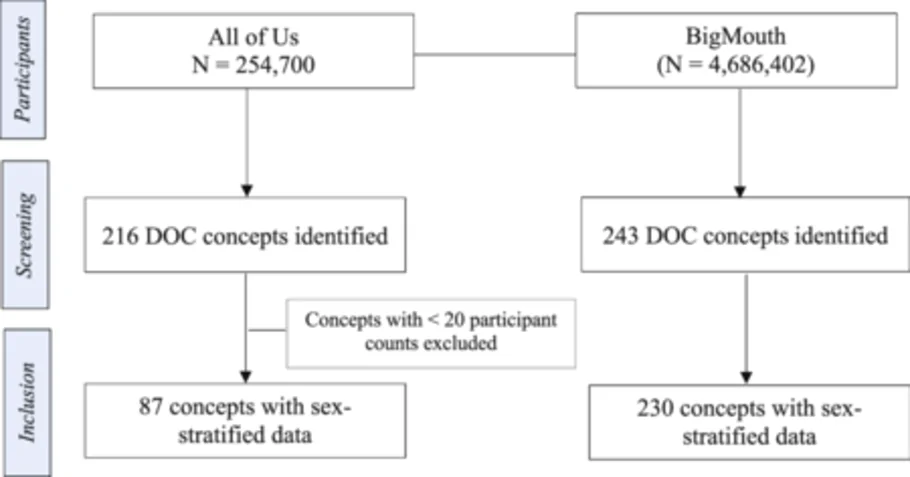
Study workflow for the inclusion of dental, oral, and craniofacial concepts investigated.

The participant count for each DOC concept in each database was then recorded and stratified by biological sex (male, female, or other). By default, exact participant counts for EHR concepts with fewer than 20 participants are not provided in *All of Us to* protect participant privacy; therefore, comparisons in such cases were not performed. Similarly, concepts showing fewer than 10 participants were not included in the analysis. The study workflow is presented in Figure 1.

### 2.2 Statistical Analysis

Sex‐specific association analysis for each DOC disease concept was performed using chi‐square tests, with a Bonferroni‐corrected *p *≤ 0.0002 to correct for multiple testing. Female‐to‐male odds ratio (OR) and 95% confidence intervals (95% CI) were also calculated to compare the relative odds of the occurrence of each concept in females and males. All statistical analyses were performed using the free online tool SISA (Simple Interactive Statistical Analysis, https://www.quantitativeskills.com/sisa/).

## 3 Results

### 3.1 Sex Differences in DOC Concepts From All of Us

A total of 216 DOC concepts were identified in *All of Us*, of which 87 had sex‐stratified frequency data. Of these, significant sex‐related differences were found for 61/87 concepts (70%), with 33 concepts (54%) showing female bias and 28 (46%) showing male bias (*p *≤ 0.0002). The top 3 concepts showing significant statistical evidence of female bias were “diseases of the oral soft tissues” [recurrent aphtous stomatitis (*p *≤ 0.0001; OR = 5.37), aphtous ulcer of mouth (*p *≤ 0.0001; OR = 5.36), xerostomia (*p *≤ 0.0001; OR = 4.96)], followed by “disorders of tooth development and eruption” [anodontia (*p *≤ 0.0001; OR = 5.29), disturbance of tooth eruption or exfoliation (*p *≤ 0.0001; OR = 5.14), dental arch relationship (*p *≤ 0.0001; OR = 4.00)], and “diseases of pulp and periapical tissues” [dental abscess (*p*≤ 0.0001; OR = 2.78), reversible pulpitis (*p*≤ 0.0001; OR = 2.68), toothache *p *≤ 0.0001; OR = 2.56)]. For “gingival and periodontal diseases,” aggressive periodontitis showed the strongest statistical evidence of female bias (*p *≤ 0.0001; OR = 2.56), followed by chronic periodontitis (*p *≤ 0.0001; OR = 1.33), and accretion of teeth (*p *≤ 0.0001; OR = 1.29). “Dental caries” and “dental caries on smooth surface penetrating into dentin” also showed statistical evidence of female bias (*p *≤ 0.0001; OR = 1.33 and OR = 1.29, respectively). Conditions in the “others” category showing significant female bias were unsatisfactory tooth restoration (*p *≤ 0.0001; OR = 4.00), and alveolitis of jaw (*p *≤ 0.0001; OR = 3.45) (Table 1).

**Table 1:** Dental, oral, and craniofacial (DOC) concepts extracted from *All of Us* showing statistically significant sex differences.

EHR concepts^a^	Participants	Females		Males		Others^b^, ^c^	*p* value	OR	95% CI	Sex bias
	*N*	*N*	%	*N*	%					
Diseases of pulp and periapical tissues										
Infection of tooth	13,060	7340	56	5720	44	360	< 0.0001	1.65	1.57–1.73	F > M
Pulpitis	1460	820	56	640	44	40	< 0.0001	1.64	1.42–1.90	F > M
Symptomatic apical periodontitis	1320	740	56	580	44	60	< 0.0001	1.63	1.40–1.90	F > M
Reversible pulpitis	580	360	62	220	38	< 20	< 0.0001	2.68	2.11–3.40	F > M
Asymptomatic apical periodontitis	580	340	59	240	41	< 20	< 0.0001	2.00	1.59–2.54	F > M
Chronic apical abscess	440	180	41	260	59	< 20	< 0.0001	0.48	0.37–0.63	M > F
Pulp and periapical tissue disease	360	140	39	220	61	< 20	< 0.0001	0.41	0.30–0.55	M > F
Toothache	260	160	62	100	38	< 20	< 0.0001	2.56	1.80–3.65	F > M
Radicular cyst	200	120	60	80	40	< 20	0.0001	2.25	1.51–3.36	F > M
Dental abscess	160	100	63	60	38	< 20	< 0.0001	2.78	1.77–4.37	F > M
Gingival and periodontal diseases										
Chronic periodontitis	4440	2380	54	2060	46	120	< 0.0001	1.33	1.23–1.45	F > M
Accretion on teeth	3460	1840	53	1620	47	100	< 0.0001	1.29	1.17–1.42	F > M
Generalized chronic periodontitis	1140	440	39	700	61	40	< 0.0001	0.40	0.33–0.47	M > F
Localized periodontitis	540	160	30	380	70	< 20	< 0.0001	0.18	0.14–0.23	M > F
Localized chronic periodontitis	540	160	30	380	70	< 20	< 0.0001	0.18	0.14–0.23	M > F
Acute periodontitis	480	200	42	280	58	< 20	< 0.0001	0.51	0.39–0.66	M > F
Generalized moderate periodontitis	300	40	13	260	87	< 20	< 0.0001	0.02	0.02–0.04	M > F
Aggressive periodontitis	260	160	62	100	38	< 20	< 0.0001	2.56	1.80–3.65	F > M
Localized moderate chronic periodontitis	220	40	18	180	82	< 20	< 0.0001	0.05	0.03–0.08	M > F
Loss of teeth due to local periodontal disease	140	40	29	100	71	< 20	< 0.0001	0.16	0.10–0.27	M > F
Complete edentulism due to periodontal disease	140	40	29	100	71	< 20	< 0.0001	0.16	0.10–0.27	M > F
Partial edentulism due to periodontal disease	140	40	29	100	71	< 20	< 0.0001	0.16	0.10–0.27	M > F
Dental caries										
Dental caries	13,000	7300	56	5700	44	360	< 0.0001	1.64	1.56–1.72	F > M
Dental caries on smooth surface penetrating into dentin	1980	1080	55	900	45	60	< 0.0001	1.44	1.27–1.63	F > M
Carious exposure of pulp	1080	460	43	620	57	40	< 0.0001	0.55	0.46–0.65	M > F
Dental caries on pit and fissure surface penetrating into dentin	720	140	19	580	81	< 20	< 0.0001	0.06	0.45–0.08	M > F
Dental caries on pit and fissure surface limited to enamel	600	160	27	440	73	< 20	< 0.0001	0.13	0.10–0.17	M > F
Dental caries on smooth surface penetrating into pulp	520	180	35	340	65	< 20	< 0.0001	0.28	0.22–0.36	M > F
Arrested dental caries	420	80	19	340	81	< 20	< 0.0001	0.06	0.04–0.08	M > F
Dental caries on pit and fissure surface penetrating into pulp	420	100	24	320	76	< 20	< 0.0001	0.10	0.07–0.13	M > F
Dental caries on smooth surface limited to enamel	120	40	33	80	67	< 20	< 0.0001	0.25	0.14–0.43	M > F
Malignant tumor of oral cavity										
Malignant tumor of oral cavity	820	340	41	480	59	40	< 0.0001	0.50	0.41–0.61	M > F
Malignant tumor of tongue	400	120	30	280	70	< 20	< 0.0001	0.18	0.14–0.25	M > F
Diseases of oral soft tissues, oral mucosa, tongue, and salivary glands										
Oral infection	13,960	8020	57	5940	43	380	< 0.0001	1.82	1.74–1.92	F > M
Candidiasis of mouth	4820	3280	68	1540	32	120	< 0.0001	4.54	4.16–4.94	F > M
Ulcer of mouth	3980	2680	67	1300	33	100	< 0.0001	4.25	3.87–4.67	F > M
Stomatitis	3720	2520	68	1200	32	80	< 0.0001	4.41	4.00–4.86	F > M
Xerostomia	2840	1960	69	880	31	80	< 0.0001	4.96	4.43–5.55	F > M
Aphthous ulcer of mouth	2320	1620	70	700	30	80	< 0.0001	5.36	4.72–6.07	F > M
Recurrent aphthous stomatitis	1460	1020	70	440	30	40	< 0.0001	5.37	4.59–6.30	F > M
Oral ulcerative mucositis due to antineoplastic therapy	400	240	60	160	40	< 20	< 0.0001	2.25	1.70–2.99	F > M
Cyst of oral soft tissue	380	240	63	140	37	< 20	< 0.0001	2.94	2.19–3.95	F > M
Enteroviral vesicular stomatitis	200	120	60	80	40	< 20	0.0001	2.25	1.51–3.36	F > M
Geographic tongue	180	120	67	60	33	< 20	< 0.0001	4.00	2.58–6.20	F > M
Disorders of Tooth Development										
Tooth disorder	18,960	11,080	58	7880	42	500	< 0.0001	1.98	1.90–2.06	F > M
Disturbance of tooth eruption or exfoliation	1960	1360	69	600	31	60	< 0.0001	5.14	4.49–5.89	F > M
Anomaly of tooth position	1640	1020	62	620	38	40	< 0.0001	2.71	2.35–3.12	F > M
Anodontia	660	460	70	200	30	< 20	< 0.0001	5.29	4.18–6.69	F > M
Retained dental root	620	200	32	420	68	< 20	< 0.0001	0.23	0.18–0.29	M > F
Dental arch relationship anomaly	120	80	67	40	33	< 20	< 0.0001	4.00	2.34–6.84	F > M
Others^c^										
Cracked tooth	1340	540	40	800	60	40	< 0.0001	0.46	0.39–0.53	M > F
Foreign body in mouth	3520	1880	53	1640	47	100	< 0.0001	1.31	1.20–1.44	F > M
Unsatisfactory tooth restoration	660	440	67	220	33	< 20	< 0.0001	4.00	3.18–5.03	F > M
Excessive attrition of teeth	540	200	37	340	63	< 20	< 0.0001	0.35	0.27–0.44	M > F
Hereditary motor and sensory neuropathy	520	220	42	300	58	< 20	< 0.0001	0.54	0.42–0.69	M > F
Alveolitis of jaw	400	260	65	140	35	< 20	< 0.0001	3.45	2.58–4.61	F > M
Abrasion of tooth	400	140	35	260	65	< 20	< 0.0001	0.29	0.22–0.39	M > F
Dental restoration failure of marginal integrity	340	80	24	260	76	< 20	< 0.0001	0.09	0.07–0.13	M > F
Contour of existing restoration of tooth biologically incompatible with oral health	200	60	30	140	70	< 20	< 0.0001	0.18	0.12–0.28	M > F
Open fracture of tooth	200	120	60	80	40	< 20	0.0001	2.25	1.51–3.36	F > M
Dental restoration esthetically inadequate or displeasing	160	40	25	120	75	< 20	< 0.0001	0.11	0.07–0.18	M > F

^a^ Based on ICD‐10‐CM as reported in All of Us (*N *= 254,700).

^b^ All of Us privacy requirements restrict knowledge of exact participant numbers when there is less than 20 in each disease concept. Not included in the total participant count.

^c^ Oral and dental disease concepts are categorized as “Others” when they constitute the sole entry within their corresponding ICD‐10‐CM category.

Abbreviations: OR: female‐to‐male odds ratio; CI: confidence interval; F: females; M: males.

In contrast, the 28 concepts exhibiting a male bias reflect a majority of concepts in “gingival and periodontal diseases” [generalized moderate periodontitis (*p *≤ 0.0001; female‐to‐male OR = 0.02), localized moderate periodontitis (*p *≤ 0.0001; OR = 0.05), and tooth loss due to local periodontal disease (*p *≤ 0.0001; female‐to‐male OR = 0.16)], followed by “dental caries” concepts [dental caries on pit and fissure penetrating into dentin (*p *≤ 0.0001; female‐to‐male OR = 0.06), dental caries on pit and fissure penetrating into pulp (*p *≤ 0.0001; female‐to‐male OR = 0.10)]. Male bias was also observed for “malignant tumor of oral cavity” concepts (*p *≤ 0.0001; female‐to‐male OR = 0.18) (Table 1).

Additional concepts showing significant sex bias in All of Us data are shown in Table 1. Concepts in which a trend for significance (0.0002 ≤ *p* ≤ 0.05) for sex bias or non‐significant results were observed are shown in Supporting Information S2: Table S1.

### 3.2 Sex Differences in DOC Concepts From BigMouth

We used BigMouth to validate findings obtained via *All of Us*. Using the same search criteria, we identified 243 oral and dental disease concepts in BigMouth that were mostly related to the concepts identified in *All of Us*. Of these, 230 concepts had sex‐specific data, with 90/230 (39%) showing statistically significant differences among the sexes (*p* ≤ 0.0002); 87 (97%) showing female bias and 3 (3%) showing male bias (*p *≤ 0.0002) (Table 2). The top 3 concepts showing a predominantly female bias were “disorders of tooth development and eruption” [cementum defect (*p *≤ 0.0001; OR = 5.39), dentin defect (*p *≤ 0.0001; OR = 3.37), enamel defect (*p *≤ 0.0001; OR = 2.56)], “diseases of the oral soft tissues, oral mucosa, tongue, and salivary glands” [xerostomia (*p *≤ 0.0001; OR = 5.93), red‐blue lesion (*p *≤ 0.0001; OR = 4.47), tongue swelling/pathology (*p *≤ 0.0001; OR = 3.61)], and “diseases of pulp and periapical tissues” [symptomatic apical periodontitis (*p *≤ 0.0001; OR = 2.77), symptomatic reversible pulpitis (*p *≤ 0.0001; OR = 2.64), and previously treated teeth (*p *≤ 0.0001; OR = 2.45)]. For “disorders of teeth, hard tissues of teeth, and supporting structures,” significant female bias was observed for temporomandibular disorders (*p *≤ 0.0001; OR = 9.72), sensitive dentin (*p *≤ 0.0001; OR = 3.40), and primary occlusal trauma (*p *≤ 0.0001; OR = 3.31). Other conditions predominantly showing female bias were harmful oral habits (*p *≤ 0.0001; OR = 2.34), systemic disease manifestation in jaw (*p *≤ 0.0001; OR = 2.19)], and benign odontogenic pathology (*p *≤ 0.0001; OR = 2.10)] (Table 2).

**Table 2:** Dental, oral, and craniofacial concepts in BigMouth showing sex bias.

EHR concept^a^	Participants	Females		Males		Others^b^	*p* value c	OR	95% CI	Sex bias
	*N*	*N*	%	*N*	%					
Diseases of pulp and periapical tissues										
Periapical diagnosis	20,310	11,818	58	8284	41	208	< 0.0001	2.02	1.94–2.10	F > M
Pulp necrosis	19,296	10,288	53	8802	46	206	< 0.0001	1.36	1.31–1.42	F > M
Previously endodontically treated	14,414	8734	61	5553	39	127	< 0.0001	2.45	2.34–2.57	F > M
Symptomatic apical periodontitis	9818	6072	62	3626	37	120	< 0.0001	2.77	2.61–2.93	F > M
Asymptomatic apical periodontitis	4294	2403	56	1858	43	33	< 0.0001	1.67	1.53–1.81	F > M
Asymptomatic irreversible pulpitis	3789	2077	55	1678	44	34	< 0.0001	1.53	1.39–1.67	F > M
Normal apical tissues	3791	2187	58	1571	41	33	< 0.0001	1.93	1.76–2.11	F > M
Chronic apical abscess	3398	1816	53	1556	46	26	< 0.0001	1.36	1.24–1.49	F > M
Previously initiated therapy	2949	1775	60	1144	39	30	< 0.0001	2.39	2.15–2.65	F > M
Normal pulp	2589	1440	56	1118	43	31	< 0.0001	1.65	1.48–1.84	F > M
Acute apical abscess	2379	1271	53	1092	46	16	< 0.0001	1.35	1.21–1.52	F > M
Symptomatic reversible pulpitis	1699	1044	61	640	38	15	< 0.0001	2.64	2.30–3.03	F > M
Asymptomatic reversible pulpitis	956	528	55	418	44	10	< 0.0001	1.59	1.33–1.90	F > M
Gingival and periodontal diseases										
Chronic periodontitis	12,7902	63,629	50	60,270	47	4003	< 0.0001	1.11	1.09–1.13	F > M
Generalized slight chronic periodontitis	59,390	28,678	48	27,080	46	3632	< 0.0001	1.11	1.09–1.14	F > M
Plaque‐induced gingival disease without local contributing factors	38,113	21,295	56	16,587	44	231	< 0.0001	1.64	1.60–1.69	F > M
Generalized moderate chronic periodontitis	31,787	16,251	51	15,387	48	149	< 0.0001	1.12	1.08–1.15	F > M
Periodontal health	30,215	16,967	56	13,183	44	65	< 0.0001	1.65	1.60–1.71	F > M
Generalized severe chronic periodontitis	22,199	10,248	46	11,838	53	113	< 0.0001	0.75	0.72–0.78	M > F
Plaque‐induced gingival disease with local contributing factors	20,461	11,304	55	9091	44	66	< 0.0001	1.54	1.49–1.61	F > M
Localized moderate chronic periodontitis	19,096	10,343	54	8678	45	75	< 0.0001	1.42	1.36–1.48	F > M
Localized severe chronic periodontitis	15,029	7649	51	7326	49	54	0.0002	1.09	1.04–1.14	F > M
Healthy periodontium with attachment loss	14,764	7695	52	7042	48	27	< 0.0001	1.19	1.14–1.25	F > M
Localized slight chronic periodontitis	6204	3502	56	2684	43	18	< 0.0001	1.20	1.14–1.25	F > M
Gingival soft tissue recession—facial and lingual surfaces	1594	946	59	647	41	1	< 0.0001	2.14	1.86–2.46	F > M
Gingival diagnosis non‐plaque induced	1489	926	62	563	38	0	< 0.0001	2.71	2.33–3.14	F > M
Mucogingival deformity—lack of keratinized gingiva	813	491	60	322	40	0	< 0.0001	2.33	1.91–2.84	F > M
Gingival swelling	787	473	60	310	39	4	< 0.0001	2.32	1.89–2.84	F > M
Insufficient biological width	626	402	64	224	36	0	< 0.0001	3.22	2.56–4.06	F > M
Inflammatory jaw lesion	536	306	57	224	42	6	< 0.0001	1.85	1.45–2.36	F > M
Gingivitis associated with lichen planus	449	349	78	100	22	0	< 0.0001	12.18	8.89–6.68	F > M
Lack of gingiva/keratinized tissue	425	267	63	158	37	0	< 0.0001	2.86	2.16–3.77	F > M
Localized slight aggressive periodontitis	192	114	59	76	40	2	0.0001	2.23	1.48–3.36	F > M
Gingival hyperplasia	166	103	62	62	37	1	< 0.0001	2.74	1.76–4.28	F > M
Gingivitis associated with pemphigoid	119	85	71	34	29	0	< 0.0001	6.25	3.56–10.97	F > M
Other soft tissue enlargement	109	72	66	36	33	1	0.000001	3.95	2.25–6.93	F > M
Gingivitis of genetic origin	109	69	63	40	37	0	0.0001	2.98	1.72–5.16	F > M
Gingivitis associated with mucocutaneous disorder	106	75	71	31	29	0	< 0.0001	5.85	3.24–10.58	F > M
Pyogenic granuloma of gingiva	82	53	65	28	34	1	0.0001	3.53	1.85–6.70	F > M
Gingival excess‐inconsistent gingival margin	68	45	66	23	34	0	0.0002	3.83	1.88–7.79	F > M
Refractory periodontitis	35	10	29	25	71	0	0.0005	0.16	0.06–0.45	M > F
Gingivitis associated with pemphigus vulgaris	34	25	74	9	26	0	0.0002	7.72	2.63–22.66	F > M
Dental caries										
Caries risk	87,845	46,858	53	40,553	46	434	< 0.0001	1.33	1.31–1.36	F > M
Caries risk high	65,653	34,577	53	30,754	47	322	< 0.0001	1.26	1.24–1.29	F > M
Primary caries (at DEJ)	57,537	30,957	54	26,192	46	388	< 0.0001	1.39	1.36–1.43	F > M
Primary caries (< 1/2 distance to the pulp)	39,646	21,339	54	17,919	45	388	< 0.0001	1.41	1.37–1.45	F > M
Recurrent caries	30,655	17,320	56	13,206	43	128	< 0.0001	1.72	1.66–1.77	F > M
Caries risk medium	20,416	11,038	54	9292	46	86	< 0.0001	1.41	1.36–1.47	F > M
Primary caries (> 1/2 distance to the pulp)	19,362	10,035	52	9170	47	157	< 0.0001	1.20	1.15–1.24	F > M
Other dental caries	141,01	7303	52	6631	47	167	< 0.0001	1.21	1.16–1.27	F > M
Recurrent caries (< 1/2 distance to the pulp)	12,099	6850	57	5208	43	41	< 0.0001	1.73	1.64–1.82	F > M
Dental caries, unspecified	10,733	5555	52	5044	47	134	< 0.0001	1.21	1.15–1.28	F > M
Caries risk low	9576	5255	55	4291	45	30	< 0.0001	1.50	1.42–1.59	F > M
Root caries	6447	3028	47	3390	53	29	< 0.0001	0.80	0.75–0.86	M > F
Recurrent caries (> 1/2 the distance to the pulp)	5235	2895	55	2305	44	35	< 0.0001	1.57	1.46–1.70	F > M
Incipient caries in enamel greater than 1/2 distance to DEJ	4933	2693	55	2189	44	51	< 0.0001	1.51	1.39–1.63	F > M
Recurrent caries (secondary caries)—(to the pulp)	2374	1283	54	1073	45	18	< 0.0001	1.43	1.27–1.60	F > M
Recurrent caries (secondary caries)—enamel	1958	1183	60	772	39	3	< 0.0001	2.35	2.06–2.67	F > M
Caries risk extreme	1869	1006	54	858	46	5	< 0.0001	1.37	1.21–1.56	F > M
Incipient caries in enamel unknown depth—NOS	681	376	55	304	45	1	0.0001	1.53	1.23–1.89	F > M
Malignant tumor of oral cavity										
Malignant neoplasm	616	258	42	358	58	0	< 0.0001	0.52	0.41–0.65	M > F
Diseases of the oral soft tissues, oral mucosa, tongue, and salivary glands										
Soft tissue calcification	2141	1143	53	983	46	15	< 0.0001	1.35	1.19–1.52	F > M
White‐yellow lesion	1791	1042	58	744	42	5	< 0.0001	1.96	1.71–2.24	F > M
Red‐blue lesion	1009	684	68	323	32	2	< 0.0001	4.47	3.71–5.39	F > M
Tongue swelling/pathology	892	583	65	306	34	3	< 0.0001	3.61	2.97–4.39	F > M
Ulcerative condition	682	405	59	276	40	1	< 0.0001	2.15	1.73–2.67	F > M
xerostomia	577	409	71	168	29	0	< 0.0001	5.93	4.60–7.64	F > M
Vesiculobullous condition	263	158	60	104	40	1	< 0.0001	2.30	1.62–3.26	F > M
Buccal mucosal swelling	256	154	60	101	39	1	< 0.0001	2.32	1.63–3.30	F > M
Oral/perioral pigmentation	198	125	63	72	36	1	< 0.0001	3.00	1.99–4.51	F > M
Disorders of tooth Development and eruption										
Abnormalities of teeth	67,895	32,882	48	30,977	46	4036	< 0.0001	1.12	1.10–1.14	F > M
Occlusion disorders	16,357	9052	55	6830	42	475	< 0.0001	1.73	1.65–1.81	F > M
Eruption abnormalities	8663	4575	53	3670	42	418	< 0.0001	1.52	1.43–1.62	F > M
Developed/acquired defects	5454	3161	58	2282	42	11	< 0.0001	1.92	1.78–2.07	F > M
Impacted tooth	4429	2335	53	1713	39	381	< 0.0001	1.77	1.63–1.92	F > M
Enamel defect	2830	1739	61	1086	38	5	< 0.0001	2.56	2.30–2.85	F > M
Dentin defect	2537	1636	64	889	35	12	< 0.0001	3.37	3.00–3.78	F > M
Enamel and dentin defect	441	264	60	176	40	1	< 0.0001	2.25	1.72–2.94	F > M
Size alteration	438	248	57	187	43	3	< 0.0001	1.75	1.34–2.29	F > M
Cementum defect	88	61	69	26	30	1	< 0.0001	5.39	2.83–10.26	F > M
Other disorders of teeth, hard tissues of teeth, and supporting structures										
Loss of tooth structure	77,435	40,573	52	36,479	47	383	< 0.0001	1.24	1.22–1.27	F > M
Sensitive dentin	2498	1614	65	872	35	12	< 0.0001	3.40	3.03–3.82	F > M
Temporomandibular disorders	1725	1304	76	417	24	4	< 0.0001	9.72	8.31–11.35	F > M
Abrasion of teeth	1221	658	54	556	46	7	< 0.0001	1.40	1.19–1.64	F > M
Abrasion of teeth due to dentifrice	435	246	57	189	43	0	0.0001	1.69	1.30–2.22	F > M
Primary occlusal trauma	420	271	65	149	35	0	< 0.0001	3.31	2.49–4.39	F > M
Secondary occlusal trauma	374	232	62	142	38	0	< 0.0001	2.67	1.99–3.59	F > M
Others^d^										
Harmful oral habits	5863	3536	60	2311	39	16	< 0.0001	2.34	2.17–2.52	F > M
Deposits on/staining of teeth	2042	1134	56	897	44	11	< 0.0001	1.59	1.19–1.34	F > M
Systemic disease manifestation in jaw	367	219	60	148	40	0	< 0.0001	2.19	1.63–2.94	F > M
Lip swelling	335	198	59	133	40	4	< 0.0001	2.20	1.61–2.99	F > M

Abbreviations: OR: female‐to‐male odds ratio; CI: confidence interval; F: female; M: male.

^a^ Reported as shown in BigMouth (total *N* = 4,686,402).

^b^ Others includes number of participants with self‐reported “other gender” and “gender not reported.”

^c^ *p* values ≤ 0.002 indicate statistical significance after Bonferroni correction; *p* values between 0.003 and 0.05 indicate a trend for significance.

^d^ Oral and dental disease concepts constituting the sole entry within their corresponding ICD‐10‐CM category in BigMouth.

Concepts showing significant statistical evidence of male bias were malignant neoplasm (*p *≤ 0.0001; female‐to‐male OR = 0.52), generalized severe aggressive periodontitis (*p *≤ 0.0001; female‐to‐male OR = 0.75), root caries (*p *≤ 0.0001; female‐to‐male OR = 0.80) (Table 2). Additional concepts showing significant sex bias in BigMouth data are shown in Table 2. Concepts in which a trend for significance (0.0002 ≤ *p* ≤ 0.05) for sex bias or non‐significant results were observed are shown in Supporting Information S2: Table S2.

### 3.3 Concepts Showing Sex Differences in Both All of Us and BigMouth

Among the concepts investigated in *All of Us* and BigMouth, complete term overlap was noted for 20 concepts, with 8 concepts showing discordant sex bias, namely “pulp degeneration/necrosis,” “chronic apical abscess,” “generalized moderate periodontitis,” “localized chronic periodontitis,” “dental caries penetrating into pulp,” “developmental abnormality of tooth size/form,” “attrition of teeth,” and “abrasion of teeth” (Table 3).

**Table 3:** DOC concepts overlapping in All of Us and BigMouth showing sex bias.

DOC concept	All of Us			BigMouth		
	Sex Bias	OR	95% CI	Sex Bias	OR	95% CI
Diseases of pulp and periapical tissues						
Dental abscess	F > M	2.78	1.77–4.37	F > M	1.35	1.21–1.52
Reversible pulpitis	F > M	2.68	2.11–3.40	F > M	1.59	1.33–1.90
Asymptomatic apical periodontitis	F > M	2.00	1.59–2.54	F > M	1.67	1.53–1.81
Symptomatic apical periodontitis	F > M	1.63	1.40–1.90	F > M	2.77	2.61–2.93
Pulp degeneration/Pulp necrosis	**M** > **F**	**0.44**	**0.25–0.78**	**F** > **M**	**1.36**	**1.31–1.42**
Chronic apical abscess	**M** > **F**	**0.48**	**0.37–0.63**	**F** > **M**	**1.36**	**1.24–1.49**
Gingival and periodontal diseases						
Generalized moderate periodontitis	**M** > **F**	**0.02**	**0.02–0.04**	**F** > **M**	**1.12**	**1.08–1.15**
Localized chronic periodontitis	**M** > **F**	**0.18**	**0.14–0.23**	**F** > **M**	**1.42**	**1.36–1.48**
Chronic periodontitis	F > M	1.33	1.23–1.45	F > M	1.11	1.09–1.13
Dental caries						
At high risk for dental caries	F > M	2.25	1.28–3.96	F > M	1.33	1.31–1.36
Dental caries	F > M	1.64	1.56–1.72	F > M	1.21	1.15–1.28
Dental caries penetrating the pulp	**M** > **F**	**0.55**	**0.46–0.65**	**F** > **M**	**1.10**	**1.03–1.18**
Malignant tumor of oral cavity						
Malignant tumor/neoplasm of oral cavity	M > F	0.50	0.41–0.61	M > F	0.52	0.41–0.65
Diseases of the oral soft tissues, oral mucosa, tongue, and salivary glands						
Xerostomia	F > M	4.96	4.43–5.55	F > M	4.25	3.87–4.67
Ulcerative condition of the mouth	F > M	4.25	3.87–4.67	F > M	2.15	1.73–2.67
Disorders of tooth development and eruption						
Disturbances in tooth eruption	F > M	5.14	4.49–5.89	F > M	1.52	1.43–1.62
Developmental abnormality of tooth size/form	**M** > **F**	**0.44**	**0.25–0.78**	**F** > **M**	**1.75**	**1.34–2.29**
Tooth disorder/abnormalities	F > M	1.98	1.90–2.06	F > M	1.12	1.10–1.14
Other disorders of teeth, hard tissues of teeth, and supporting structures						
Attrition of teeth	**M** > **F**	**0.35**	**0.27–0.44**	**F** > **M**	**1.09**	**1.03–1.16**
Abrasion of teeth	**M** > **F**	**0.29**	**0.22–0.39**	**F** > **M**	**1.40**	**1.19–1.64**

*Note:* Bold font denotes differences in sex bias among the results from the two databases.

Abbreviations: OR, female‐to‐male odds ratio; CI, confidence Interval.

## 4 Discussion

In the present study, we compared the frequencies of more than 200 DOC disease concepts among 4,501,648 females and males residing in the United States utilizing two large data repositories (*All of Us* and BigMouth) featuring electronic health record data. Among the DOC disease concepts included in the analysis, significant sex‐specific differences were found for 70% and 39% of the concepts found in *All of Us* and BigMouth, respectively. Specifically, a female predominance was identified in 54% and 97% of these dental concepts in each database. Instances of discordant sex bias among concepts in each database were also observed, albeit minimal.

Among the seven general concept categories in the *All of Us* database, “disorders of tooth development and eruption,” “diseases of the oral soft tissues, oral mucosa, tongue, and salivary glands,” and “diseases of pulp and periapical tissues” were marked by female bias, meanwhile “gingival and periodontal diseases,” “malignant tumors of the oral cavity,” “dental caries,” and “other” disorders were marked by male bias. Periodontal disease is widely known to affect males more frequently than females. A systematic review (Shiau and Reynolds 2010) and two US population‐based studies (Albandar 2002) have confirmed a significantly male predilection in periodontal disease, with effect sizes ranging from 7.33 to 16.85. Potential explanations for this well‐established sex difference may be attributed to biological, immunological, and behavioral reasons. For instance, sexual dimorphism has been reported to account for a more diverse oral microbiome, with males presenting an increased abundance of *Prevotella intermedia* compared to females (Vieira et al. 2017). Males also tend to demonstrate a more robust intrinsic inflammatory reaction to both injuries and microbial pathogens compared to females (Shiau and Reynolds 2010). This is evidenced by an increased toll‐like receptor (TLR)‐4 signaling, distinct cytokine production (Klein and Flanagan 2016), and greater CD8 + T cell count (Abdullah et al. 2012) in males. Moreover, risk factors commonly associated with periodontal disease (e.g., obesity, cardiovascular disease, type II diabetes), are also known to exhibit a male bias (Kim and Shin 2020; Meisel et al. 2014; Regensteiner et al. 2015). Lastly, a study by Lipsky et al. (2021) suggested that men are less inclined to prioritize their oral health, therefore increasing the potential to higher instances of periodontal disease. In contrast, “gingival and periodontal diseases” in BigMouth were marked by female bias. Interestingly, these reflected specific gingivitis conditions and periodontitis manifestations frequently associated with autoimmune conditions. The observed higher female predilection could potentially be attributed to hormonal effects during pregnancy or menstrual cycles, as well as a more robust innate and adaptive immune response that makes females more vulnerable to autoimmune and anti‐inflammatory conditions (Klein and Flanagan 2016).

Disorders of teeth, hard tissues of teeth, and supporting structures were also more prevalent in males from *All of Us*. Many of these included loss of teeth due to periodontal disease, which may result from a male bias for periodontal disease. Nevertheless, BigMouth data revealed a higher prevalence of these concepts in females. A possible explanation for such a discrepancy is that the terminology for the dental concepts identified within the two databases examined were not completely standardized, and BigMouth often contained additional subtypes of each concept compared to *All of Us*. There might also be an inherent bias in BigMouth as the participants included mostly females.

Dental caries concepts exhibited a female bias in both databases, with females presenting between 1.3‐ and 1.64‐fold higher risk of overall dental caries than males. When considering additional dental caries subtypes in *All of Us*, however, a marked male bias was observed for 7/9 concepts including carious exposure of pulp, dental caries on pit and fissure (in enamel, into dentin, and into pulp). In BigMouth, of the 18 dental caries‐associated concepts, only root caries showed male bias. Female bias in dental caries has been supported by several narrative and systematic reviews (Lukacs and Largaespada 2006; Ferraro and Vieira 2010; Teshome et al. 2021). Moreover, an earlier tooth eruption in females, states of pregnancy, among others, have been suggested as potential reasons for a higher female prevalence for dental caries (Lukacs and Largaespada 2006). Additional studies support a similar prevalence of dental caries among the sexes (Shaffer et al. 2015; Skoskiewicz‐Malinowska et al. 2021).

With the exception of “chronic apical abscess” and “pulp and periapical disease” that showed a male bias in All of Us, all concepts under “diseases of the pulp and periapical tissues” category showed predominantly female bias in All of Us and BigMouth. Particularly, concepts associated with dental pain, such as toothache, symptomatic reversible pulpitis, and symptomatic apical periodontitis were at least 2 times more frequent in females than males. Similar prevalence rates and instances of either higher female or male predominance in pulp and periapical diseases have been documented in the literature (Berlinck et al. 2015; Ferreira et al. 2022; Huumonen et al. 2017).

Similarly, both databases were concordant in identifying a female bias for all “diseases of oral soft tissues, tongue, and salivary glands,” with some concepts showing higher than fivefold increased odds in females compared to males. Considering that many of these conditions have an autoimmune etiology, a higher female predisposition was expected (Klein and Flanagan 2016). Disorders of tooth development and eruption as well as dentofacial anomalies were also significantly more prevalent in females compared to males, similarly to previous reports (Letra 2022).

Males displayed a significantly higher risk of oral malignancies compared to females in both databases. This has been consistently reported across various studies worldwide, where males exhibit between 1‐ to fivefold increased risk compared to females (Park et al. 2022; Ward et al. 2019). Such higher male predisposition to cancer persists even after controlling for behavioral risk factors, including smoking and alcohol consumption (Park et al. 2022). This phenomenon has been attributed to the potential association between androgens and cancer development, whereas estrogens have been proposed to exert a protective effect (Park et al. 2022).

Increasing evidence has supported genetic predisposition as a contributor to sex‐based biological differences in oral and dental diseases (Sangalli et al. 2023). In this context, female bias towards caries risk has been attributed to genetic variation in genes located on the X chromosome, which are implicated in amelogenesis, dietary preferences, oral microbiome composition, and salivary flow (Ferraro and Vieira 2010). Similarly, studies have reported that genetic polymorphisms may result in an altered host response to microbial challenges in the dental pulp, therefore modulating caries progression and/or predisposition to pulp and periapical disease (Menezes‐Silva et al. 2012; Messing et al. 2019; Petty et al. 2023). More Notably, sex‐specific associations with polymorphisms in genes involved in inflammation, wound healing, bone remodeling, and apoptosis, have been reported for periapical disease (de Souza et al. 2019; Petty et al. 2023). In animal models, sexual dimorphism in immunological responses to oral infectious diseases, including differences in neutrophil trafficking, osteoclast differentiation, and formation in cases of both periodontal and periapical disease, has been widely reported (Valerio and Kirkwood 2018). Sex differences have also been reported for the extent of activation of innate and adaptive immune cells in females, including higher numbers of macrophages, dendritic cells, antigen‐presenting cells, interferon activity, lymphocyte cells, CD4 + T cells, and cytotoxic T cells (Klein and Flanagan 2016). Taken together, these multiple lines of evidence strongly support a role for sex as a biological variable underlying the pathogenesis of pulpal and periapical diseases, although non‐biological factors may also play a role.

This study has numerous strengths but also has some limitations. Among the strengths is the large sample size surpassing previous studies and greatly enhancing the generalizability of our findings, as well as the large number and wide spectrum of DOC concepts investigated. Study limitations include inherent differences among the databases as each aggregates data from different sources that may contribute to occasional discrepant findings. For instance, *All of Us* contains medical and dental record data from individuals across the US who agree to share their data, whereas BigMouth data is directly imported from dental electronic health records from eleven participating dental schools. Hence, a lack of complete agreement between the participant community and concept terminologies in *All of Us* and BigMouth databases was expected. *All of Us* research program was intentionally designed to capture data from a participant cohort reflecting the diversity of the U.S. population, with intentional recruitment of populations historically underrepresented in biomedical research. By contrast, BigMouth is not a purposefully recruited research cohort but consists of electronic health record data generated through routine care delivered at participating dental institutions. Thus, demographic differences beyond sex between the two study populations may have contributed to discrepancies in the findings. Given that the prevalence and susceptibility to several DOC conditions may vary across ethnic and racial backgrounds (Russell et al. 2008; Eke et al. 2015; Centers for Disease Control and Prevention 2019), and that these factors may intersect with sex, future studies should evaluate whether sex‐related differences in DOC conditions vary across racial and ethnic groups and how these differences may affect the generalizability of our findings. *All of Us* provided broader group categories, whereas BigMouth featured more concepts and very specific subcategories based on dental diagnostic terminology. Importantly, dental data from *All of Us* is more likely to be a result of self‐report or within electronic medical records, whereas BigMouth data is retrieved from electronic dental records reflecting actual patient interactions. Among the main discordant findings of sex bias in the two databases were “pulp degeneration/necrosis,” “chronic apical abscess,” “generalized moderate and localized chronic periodontitis,” “dental caries penetrating the pulp,” “developmental anomaly of tooth size/form,” and “tooth abrasion” and “attrition.” While these differences may have precluded more accurate comparisons between the databases, the overall similarities in the findings of female or male bias across many of the concepts investigated in the two databases are invaluable in identifying consistent patterns of sex disparities in oral and dental diseases and conditions on a large scale.

## 5 Conclusions

Evidence supporting sex‐specific differences DOC diseases and conditions is still scarce. This study corroborates previous reports suggesting association of sexual dimorphism in periodontal disease, dental caries, and malignant tumors of the oral cavity meanwhile providing new evidence for sexual dimorphism in pulpal and periapical disease, disorders of oral soft tissues and tooth development, and other conditions. These findings provide valuable insight as they may be used to inform future studies aimed at uncovering the biological and non‐biological factors that underlie sex‐specific differences in disease, and towards clinical translation with sex‐specific strategies to improve oral health of females and males. Further studies considering socio‐economic and behavioral factors may also provide valuable insight into the role of sex differences in DOC diseases and conditions.

## Author Contributions

E.F. contributed to data acquisition and analysis and drafted the manuscript. L.S. contributed to data interpretation and drafted the manuscript. A.L. contributed to conception and data interpretation and critically reviewed the manuscript.

## Conflicts of Interest

The authors declare no conflicts of interest.

## Supporting information


Supporting File 1

Supporting File 2

## Data Availability

Data from the All of Us Program is publicly available. Raw data obtained in this study are available in the Supplemental material.
