# Supplementary material for: Examining Electronic Health Records to Investigate Sex Differences in Oral Diseases and Conditions
Source: Clin Exp Dent Res. 2026 Sep 8;12(5):e70455. doi: 10.1002/cre2.70455 (PMC13552865; doi:10.1002/cre2.70455)
Supplement: Supplementary file 1 [file CRE2-12-e70455-s002.docx]

**Supplementary Materials**

**Supplementary Table 1. Additional dental, oral and craniofacial concepts investigated in All of Us.**

| **EHR concepts*** | **Participant count** | **Female count** | **Male count** | **Other gender** | **P-value**^#^ | **Sex bias (trend)** |
| --- | --- | --- | --- | --- | --- | --- |
| dental caries extending into dentin | 2160 | 1040 | 980 | 60 | 0.07 | none |
| broken tooth without complication | 960 | 480 | 420 | <20 | 0.006 | F>M |
| fractured dental restorative material | 860 | 380 | 420 | <20 | 0.05 | none |
| fractured dental restorative material with loss of material | 720 | 320 | 360 | <20 | 0.03 | M>F |
| necrosis of the pulp | 700 | 320 | 360 | <20 | 0.03 | M>F |
| oral phase dysphagia | 640 | 320 | 280 | <20 | 0.03 | F>M |
| leukoplakia of oral mucosa | 600 | 280 | 300 | <20 | 0.25 | none |
| neoplasm of uncertain behavior of lip, oral cavity and pharynx | 420 | 200 | 220 | <20 | 0.17 | none |
| irreversible pulpitis | 360 | 180 | 180 | <20 | 1 | none |
| laceration of oral cavity | 320 | 140 | 140 | <20 | 1 | none |
| neoplasm of uncertain behavior of oral cavity | 240 | 120 | 120 | <20 | 1 | none |
| erosion of teeth | 220 | 100 | 120 | <20 | 0.06 | none |
| disturbance of oral epithelium | 180 | 80 | 100 | <20 | 0.04 | M>F |
| at risk for dental caries | 140 | 80 | 60 | <20 | 0.02 | F>M |
| failure of osseointegration of dental implant | 120 | 60 | 60 | <20 | 1 | none |
| dislocation of tooth | 120 | 60 | 60 | <20 | 1 | none |
| broken tooth with complication | 120 | 80 | 60 | <20 | 0.009 | F>M |
| cellulitis of oral soft tissues | 120 | 60 | 60 | <20 | 1 | none |
| pulp degeneration | 100 | 40 | 60 | <20 | 0.005 | M>F |
| developmental abnormality of tooth size and form | 100 | 40 | 60 | <20 | 0.005 | M>F |
| oral ulceration due to radiation burn | 100 | 40 | 60 | <20 | 0.005 | M>F |
| primary malignant neoplasm of floor of mouth | 100 | 40 | 60 | <20 | 0.005 | M>F |
| at high risk for dental caries | 100 | 60 | 40 | <20 | 0.005 | F>M |
| tooth loss | 80 | 40 | 40 | <20 | 1 | none |
| biological failure of dental implant after osseointegration | 60 | 40 | 40 | <20 | 1 | none |
| fracture of dental implant | 60 | 40 | 40 | <20 | 1 | none |

* EHR, electronic health records reported as shown in All of Us (total N = 254,700)

^#^ Chi-square test; P-values ≤ 0.0002 indicate statistical significance after Bonferroni correction; P-values between 0.0003 and 0.05 indicate trend for significance

F: female; M: male

**Supplementary Table 2. Additional dental, oral and craniofacial concepts investigated in BigMouth.**

| **EHR concept*** | **Participant count** | **Female count** | **Male count** | **Other gender** | **Gender not reported** | **P-value^#^** | **Sex bias (trend)** |
| --- | --- | --- | --- | --- | --- | --- | --- |
| number alteration | 53739 | 24882 | 25229 | 4 | 3617 | 0.03 | M>F |
| enamel fracture | 15793 | 7975 | 7777 | 2 | 39 | 0.02 | F>M |
| enamel and dentin fracture | 12311 | 6190 | 6052 | 3 | 63 | 0.07 | None |
| attrition of teeth | 7972 | 4065 | 3890 | 1 | 16 | 0.005 | F>M |
| primary caries to the pulp | 7521 | 3797 | 3614 | 7 | 102 | 0.003 | F>M |
| complete anodontia | 5337 | 2656 | 2673 | 1 | 6 | 0.74 | None |
| aggressive periodontitis | 4009 | 2053 | 1938 | 1 | 16 | 0.01 | F>M |
| anatomic abnormalities | 2470 | 1223 | 1220 | 1 | 26 | 1 | None |
| cervical caries | 2320 | 1134 | 1175 | 1 | 10 | 0.22 | None |
| hard tissue abnormality | 2144 | 1068 | 1050 | 1 | 25 | 0.58 | None |
| compromised crown:root ratio | 1980 | 1025 | 950 | 1 | 4 | 0.02 | None |
| crown-root fracture | 1808 | 920 | 884 | 0 | 4 | 0.23 | None |
| unspecified periodontal disease and condition | 1624 | 832 | 786 | 0 | 6 | 0.10 | None |
| rampant caries | 1554 | 763 | 769 | 0 | 22 | 0.82 | None |
| abscess of the periodontium | 1537 | 798 | 730 | 0 | 9 | 0.01 | F>M |
| erosion of teeth | 1327 | 616 | 706 | 1 | 4 | 0.0005 | M>F |
| enamel and dentin fracture with pulp involvement | 1283 | 602 | 676 | 1 | 4 | 0.003 | M>F |
| generalized moderate aggressive periodontitis | 1274 | 665 | 602 | 0 | 6 | 0.01 | F>M |
| shape alteration | 1147 | 605 | 540 | 0 | 2 | 0.006 | F>M |
| combined periodontic-endodontic lesion | 1033 | 534 | 493 | 0 | 6 | 0.07 | None |
| localized severe aggressive periodontitis | 928 | 484 | 441 | 0 | 3 | 0.04 | F>M |
| disturbances in tooth formation | 918 | 480 | 438 | 0 | 0 | 0.05 | None |
| normal periapical tissues | 888 | 470 | 410 | 1 | 7 | 0.004 | F>M |
| generalized severe aggressive periodontitis | 821 | 374 | 444 | 0 | 3 | 0.0005 | M>F |
| vertical and/or horizontal ridge deficiency | 763 | 393 | 370 | 0 | 0 | 0.24 | None |
| cyst of the oral region | 752 | 395 | 350 | 0 | 7 | 0.02 | F>M |
| localized moderate aggressive periodontitis | 723 | 373 | 349 | 0 | 1 | 0.20 | None |
| aberrant frenum/muscle position | 630 | 329 | 293 | 0 | 7 | 0.04 | None |
| incipient caries in enamel less than 1/2 way to DEJ | 629 | 310 | 317 | 0 | 2 | 0.70 | None |
| arrested dental caries | 614 | 323 | 287 | 0 | 4 | 0.03 | F>M |
| face swelling | 536 | 293 | 234 | 0 | 9 | 0.0003 | F>M |
| benign nonodontogenic path. | 480 | 245 | 234 | 0 | 1 | 0.48 | None |
| inadequate crown length | 471 | 251 | 219 | 0 | 1 | 0.04 | F>M |
| genetic jaw disease | 468 | 250 | 216 | 0 | 2 | 0.02 | F>M |
| size alteration | 438 | 248 | 187 | 0 | 3 | 0.0004 | F>M |
| external resorption | 349 | 201 | 191 | 0 | 2 | 0.44 | None |
| secondary pulpal conditions | 342 | 160 | 181 | 0 | 1 | 0.10 | None |
| soft tissue abnormality | 334 | 158 | 175 | 0 | 1 | 0.18 | None |
| caries of cementum | 298 | 142 | 156 | 0 | 0 | 0.25 | None |
| abnormal gingival color | 285 | 157 | 126 | 0 | 2 | 0.009 | F>M |
| gingival abscess | 281 | 158 | 121 | 0 | 2 | 0.002 | F>M |
| asymptomatic apical periodontitis | 256 | 127 | 128 | 0 | 1 | 1 | None |
| complete anodontia (congenital) | 255 | 129 | 125 | 0 | 1 | 0.72 | None |
| verrucal-papillary lesion | 253 | 133 | 119 | 0 | 1 | 0.21 | None |
| supernumerary teeth (including fourth molar) | 250 | 112 | 125 | 0 | 13 | 0.24 | None |
| internal resorption | 233 | 104 | 128 | 0 | 1 | 0.03 | M>F |
| paranasal sinus pathology | 232 | 121 | 109 | 0 | 2 | 0.26 | None |
| atrophy of edentulous alveolar ridge | 228 | 122 | 106 | 0 | 0 | 0.13 | None |
| irregular alveolar process | 222 | 108 | 110 | 0 | 4 | 0.85 | None |
| generalized slight aggressive periodontitis | 212 | 113 | 96 | 1 | 2 | 0.10 | None |
| fistula | 182 | 101 | 79 | 0 | 2 | 0.02 | F>M |
| benign odontogenic pathology | 174 | 102 | 70 | 0 | 2 | 0.0006 | F>M |
| horizontal root fracture (apical) | 160 | 76 | 82 | 0 | 2 | 0.50 | None |
| salivary gland pathology | 158 | 85 | 70 | 0 | 3 | 0.10 | None |
| periodontal abscess | 147 | 67 | 79 | 0 | 1 | 0.16 | None |
| skin lesion | 132 | 71 | 60 | 0 | 1 | 0.17 | None |
| mucogingival deformity- aberrant frenum/muscle position | 124 | 76 | 48 | 0 | 0 | 0.0004 | F>M |
| gingival soft tissue recession-interproximal-papillary | 107 | 53 | 54 | 0 | 0 | 1 | None |
| other hard tissue lesion | 104 | 54 | 44 | 0 | 6 | 0.16 | None |
| habitual abrasion of teeth | 103 | 50 | 53 | 0 | 0 | 0.67 | None |
| gingivitis associated with streptococcal species | 101 | 54 | 47 | 0 | 0 | 0.32 | None |
| external resorption | 93 | 45 | 48 | 0 | 0 | 0.65 | None |
| diabetes mellitus associated gingivitis | 83 | 49 | 34 | 0 | 0 | 0.02 | F>M |
| gingival soft tissue enlargement | 81 | 43 | 38 | 0 | 0 | 0.43 | None |
| pericoronal abscess | 80 | 41 | 39 | 0 | 0 | 0.75 | None |
| symptomatic apical periodontitis | 79 | 47 | 31 | 0 | 1 | 0.01 | F>M |
| palatal swelling | 72 | 36 | 35 | 0 | 1 | 0.86 | None |
| ankylosis | 68 | 35 | 32 | 0 | 1 | 0.60 | None |
| drug induced gingivitis-other | 63 | 31 | 31 | 0 | 1 | 1 | None |
| enlargement of alveolar ridge | 60 | 28 | 30 | 0 | 2 | 0.71 | None |
| gingival excess/display | 59 | 33 | 26 | 0 | 0 | 0.20 | None |
| disorder of teeth and supporting structures, unspecified | 58 | 28 | 30 | 0 | 0 | 0.71 | None |
| gingival excess-pseudopocket | 57 | 37 | 20 | 0 | 0 | 0.001 | F>M |
| other surface lesion | 53 | 29 | 24 | 0 | 0 | 0.33 | None |
| mucogingival deformity- decreased vestibular depth | 49 | 30 | 19 | 0 | 0 | 0.03 | F>M |
| gingival lesions | 49 | 29 | 20 | 0 | 0 | 0.07 | None |
| gingivitis associated w/ primary herpetic gingivostomatitis | 47 | 29 | 18 | 0 | 0 | 0.02 | F>M |
| condensing osteitis | 43 | 29 | 14 | 0 | 0 | 0.001 | F>M |
| primary occlusal trauma2 | 41 | 19 | 22 | 0 | 0 | 0.50 | None |
| periodontitis-root fracture | 40 | 21 | 19 | 0 | 0 | 0.65 | None |
| floor of mouth swelling | 39 | 17 | 22 | 0 | 0 | 0.25 | None |
| obliteration of root canal | 38 | 22 | 16 | 0 | 0 | 0.17 | None |
| gingival lesions-foreign body reaction | 38 | 25 | 13 | 0 | 0 | 0.006 | F>M |
| decreased vestibular depth | 37 | 20 | 17 | 0 | 0 | 0.48 | None |
| necrotizing periodontal dx | 37 | 18 | 19 | 0 | 0 | 0.81 | None |
| acute apical abscess | 35 | 13 | 22 | 0 | 0 | 0.03 | M>F |
| refractory periodontitis | 35 | 10 | 25 | 0 | 0 | 0.0003 | M>F |
| necrotizing ulcerative gingivitis (nug) | 33 | 18 | 15 | 0 | 0 | 0.46 | None |
| hereditary disturbances in tooth structure, no origin specified | 33 | 18 | 14 | 0 | 1 | 0.32 | None |
| periodontosis | 32 | 18 | 14 | 0 | 0 | 0.31 | None |
| pulp abnormalities | 29 | 14 | 14 | 0 | 1 | 1 | None |
| puberty associated gingivitis | 29 | 18 | 11 | 0 | 0 | 0.06 | None |
| post adolescent periodontitis | 25 | 16 | 9 | 0 | 0 | 0.05 | None |
| gingivitis associated with recurrent oral herpes | 24 | 14 | 10 | 0 | 0 | 0.25 | None |
| metabolic jaw disease | 22 | 14 | 8 | 0 | 0 | 0.07 | None |
| periodontitis-dental restorations and appliances | 22 | 14 | 8 | 0 | 0 | 0.07 | None |
| gingivitis modified by malnutrition- no origin specified | 22 | 14 | 8 | 0 | 0 | 0.07 | None |
| periodontitis-tooth anatomic factors | 20 | 12 | 8 | 0 | 0 | 0.20 | None |
| fibrous epulis | 19 | 14 | 5 | 0 | 0 | 0.003 | F>M |
| hyperplasic pulpitis | 18 | 11 | 7 | 0 | 0 | 0.18 | None |
| periodontitis-cervical root resorption and cemental tears | 18 | 11 | 7 | 0 | 0 | 0.18 | None |
| rapidly progressive periodontitis | 17 | 8 | 9 | 0 | 0 | 0.73 | None |
| dental restorative material-other | 17 | 11 | 6 | 0 | 0 | 0.08 | None |
| dental restorative material-nickel | 16 | 10 | 6 | 0 | 0 | 0.16 | None |
| flabby ridge | 16 | 8 | 8 | 0 | 0 | ------- | None |
| gingivitis associated with candida | 14 | 9 | 5 | 0 | 0 | ------- | None |
| throat/neck swelling | 13 | 9 | 4 | 0 | 0 | 0.05 | None |
| radiation caries | 13 | 8 | 5 | 0 | 0 | 0.23 | None |
| juvenile periodontitis | 12 | 7 | 5 | 0 | 0 | 0.41 | None |
| drug induced gingivitis-oral contraceptive associated | 11 | 7 | 4 | 0 | 0 | ------- | None |
| pericoronal abscess | 11 | 8 | 3 | 0 | 0 | ------- | None |
| gingivitis associated with varicella zoster | 9 | 4 | 5 | 0 | 0 | ------- | None |
| widened periodontal ligament | 8 | 5 | 3 | 0 | 0 | 0.31 | None |
| gingivitis associated with lupus erythematosus | 8 | 7 | 1 | 0 | 0 | ------- | F>M |
| gingivitis modified by malnutrition-ascorbic acid deficient | 8 | 4 | 4 | 0 | 0 | ------- | None |
| gingival fibromatosis | 8 | 2 | 6 | 0 | 0 | ------- | None |
| gingivitis associated with histoplasmosis | 7 | 6 | 1 | 0 | 0 | ------- | None |
| gingivitis associated with treponema pallidum | 7 | 4 | 3 | 0 | 0 | ------- | None |
| focal epithelial hyperplasia of mouth or tongue | 7 | 1 | 6 | 0 | 0 | ------- | None |
| dental restorative material-acrylic | 6 | 2 | 4 | 0 | 0 | ------- | None |
| dental restorative material-mercury | 6 | 4 | 2 | 0 | 0 | ------- | None |
| prepubertal periodontitis | 5 | 1 | 4 | 0 | 0 | ------- | None |
| gingivitis associated with other blood dyscrasias | 5 | 4 | 1 | 0 | 0 | ------- | None |
| mucogingival deformity-abnormal color | 4 | 3 | 1 | 0 | 0 | ------- | None |
| Pulp stones | 3 | 2 | 1 | 0 | 0 | ------- | None |
| gingivitis associated with erythema multiforme | 3 | 2 | 1 | 0 | 0 | ------- | None |

* EHR, electronic health records reported as shown in BigMouth (total N = 4,686,402); concepts with < 10 male or female counts were not included in the analysis

^#^ Chi-square test; ^#^ P-values ≤ 0.0002 indicate statistical significance after Bonferroni correction; P-values between 0.0003 and 0.05 indicate trend for significance

F: female; M: male

OR: odds ratio; CI: confidence interval

F: female; M: male
